# The effect of transcutaneous auricular vagus nerve stimulation on HRV in healthy young people

**DOI:** 10.1371/journal.pone.0263833

**Published:** 2022-02-10

**Authors:** Duyan Geng, Xuanyu Liu, Yan Wang, Jiaxing Wang

**Affiliations:** 1 State Key Laboratory of Reliability and Intelligence of Electrical Equipment, Hebei University of Technology, Tianjin, China; 2 Key Laboratory of Electromagnetic Field and Electrical Apparatus Reliability of Hebei Province, Hebei University of Technology, Tianjin, China; University of Pennsylvania Perelman School of Medicine, UNITED STATES

## Abstract

Transcutaneous auricular vagus nerve stimulation (taVNS) has shown positive effects on a variety of diseases. Considering that decreased heart rate variability (HRV) is closely associated with morbidity and mortality for a variety of diseases, it is important to investigate the effect of taVNS on HRV. In Study 1, we conducted a two-stage cross-over trial to compare the effects of taVNS and sham taVNS (staVNS) on HRV. In Study 2, we systematically tested the effects of different taVNS parameters on high frequency (HF) component of HRV. The results showed that taVNS significantly increased measurements of root mean square of the difference between successive RR intervals (RMSSD), percentage of number of pairs of adjacent RR intervals differing greater than 50ms (pRR50), standard deviation of all RR intervals (SDRR), HF. Significantly, enhancement of HF and pRR50 persisted into recovery period. In addition, higher baseline LF/HF ratio was associated with greater LF/HF ratio decrease. Findings also showed that there was no significant difference in measurements of HF between different taVNS parameters. These studies suggest that taVNS could increase HRV, it may help taVNS in the treatment of low HRV related diseases. However, taVNS may not have parameter-specific effects on HRV.

## Introduction

Vagus nerve (VN) is a key component of autonomic nervous system and plays an important strategic role in human body [1]. It is the longest cranial nerve and is distributed throughout various internal organs such as cardiac, liver, esophagus etc. Therefore, VN acts as a bridge between the brain and various internal organs and is involved in the regulation of multiple major systems [2]. Increased cardiac vagal activity is proportionally associated with health, wellbeing, relaxation, and even emotions like empathy, whereas decreased cardiac vagal activity relates to risk factors such as morbidity, mortality, and stress [3, 4]. Artificial modulation of afferent vagus nerve, which is a powerful entrance to the brain, affects a large number of physiological processes implicating interactions between the brain and body. VN thus plays a crucial role in determining brain-body interactions [5].

Activation of VN can be performed through implantable vagus nerve stimulator (VNS) [6]. FDA had respectively approved the treatment for epilepsy and depression with VNS in 1997 and 2005 [7, 8]. In addition, VNS is considered as a potential therapy for a wide range of conditions including heart failure, chronic pain and obesity [9–11]. However, VNS requires surgical implantation of electrodes and need to wear instruments for a long time after operation, which is at risk of infection. Besides, some side effects, such as cough and pain, limit the application of VNS [12].

TaVNS is a simple and non-invasive therapy that modulates VN by stimulating auricular branches of VN with few side effects [13, 14]. The literature in this area is growing fast due to the availability of resources and noninvasiveness. At present, taVNS is an emerging technology in the field of bioelectronic medicine with applications in therapy and has been applied in the treatment of many diseases, including neurodegenerative diseases, chronic pain diseases, inflammation, cardiovascular diseases, etc. [15]. Recently, a study showed that taVNS could be a potential treatment for Covid19-Originated acute respiratory distress syndrome [16]. However, the research on taVNS is still in the preliminary stage, and the stimulation parameters sets in related studies have not been used consistently [17], which impedes the comparability of such studies.

Heart rate variability (HRV), the variation over time of the period between consecutive heartbeats, is a reliable reflection of many physiological factors that regulate normal heart rhythm [18]. It is considered that HRV is a kind of standard method for noninvasive assessment of autonomic function and can be used to measure efferent vagus nerve activity [19]. Furthermore, decreased HRV is closely associated with morbidity and mortality for a variety of diseases, such as cardiovascular disease, autonomic nervous imbalance-related diseases, cancer, and Alzheimer’s disease [20]. Studies have shown that there are usually three physiological mechanisms contributing to the occurrence and progression of these diseases, that is, sympathetic over-activity, inflammatory response and oxidative stress [20, 21], whereas VN-mediated parasympathetic markers of HRV are significantly inversely correlated with inflammatory markers [22], oxidative stress [23] and norepinephrine [24]. Thus, it is necessary to systematically investigate the influence of taVNS on HRV, which can not only help us investigate the influence of taVNS on autonomic function, but also explore whether HRV can be used as a biomarker of taVNS, as it can help guide the research on clinical applications and can inform researchers on optimal stimulation sites and parameters to further optimize treatment efficacy.

In the first study, we compared the effects of taVNS on HRV with the effects of sham stimulation and observed whether there was a carry-over effect. We hypothesized that taVNS would produce a greater increase in HRV than sham. The second study aimed to investigate whether taVNS has parameter-specific effects on high frequency (HF) power, which is an indicator of cardiac vagal tone and usually assessed in related studies. We expected that parameters of higher energy density (larger pulse width, higher frequency) would be more effective in changing HF than lower energy combinations.

## Materials and methods

### Participants

The sample consisted of healthy college students at the local university. Participants were eligible if they did not have neurological diseases (epilepsy, migraine), cardiovascular illnesses, severe inflammation and pregnancy at the period of experiment. They were asked not to exercise, smoke, or take any drug (alcohol, caffeine) which might influence autonomic nervous system 5 hours before participation. All participants gave written informed consent.

Fourteen participants were recruited for study 1 and an additional twenty volunteers participated in study 2. All studies were approved by the ethical committee of the Hebei University of Technology and conformed to the standards outlined in the Declaration of Helsinki.

### Transcutaneous auricular vagus nerve stimulation(taVNS)

We employed the PARASYM taVNS device (Parasym Ltd, UK) as the stimulation system, which can deliver stimulation to ear targets through modified surface electrodes. Stimulation targets were cleaned by alcohol cotton swabs to remove oil from the ear surface and reduce skin resistance.

**Fig 1:**
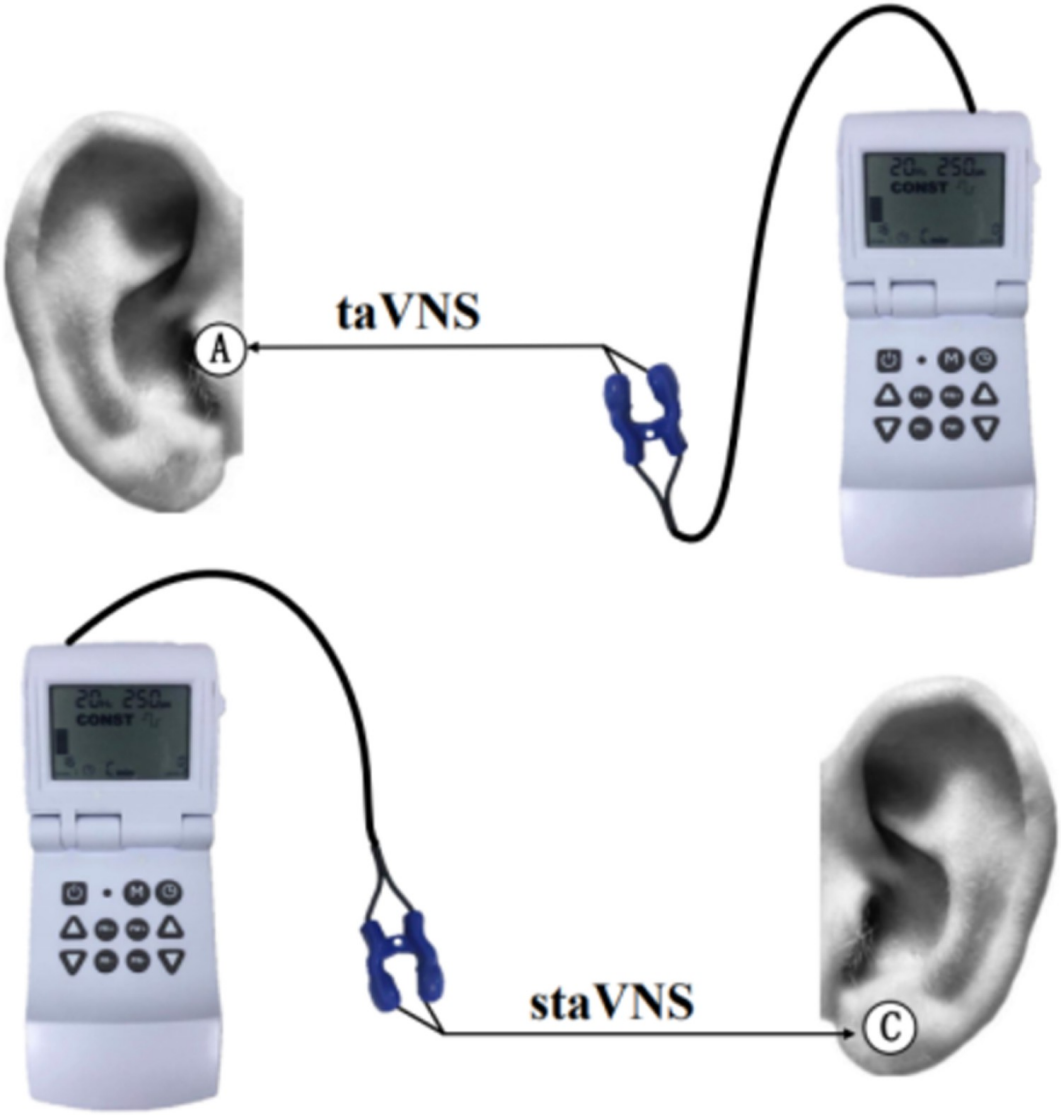
Ear stimulation targets used of taVNS or staVNS. The stimulation target of taVNS was at the A site (tragus, active site), which is rich in vagus nerve distribution. Whereas the stimulation target of staVNS was at the C site (earlobe, control site), where was no distribution of vagus nerve [25].

In study 1, the active condition was performed at the inner side of the left tragus by taVNS device with 20Hz of frequency and 250us of pulse. The stimulation intensity was adjusted to the level of the perceptual threshold (PT), which means that the stimulation intensity increased slowly from level 0 until the participants reported that they could feel the current without pain. According to prior literature and cadaver trials [25], there was no distribution of VN by the earlobe. In order to eliminate the influence of irrelevant variables (pain sensation, subjective tension emotion) on the outcome analysis, we performed sham stimulation placebo condition (sham taVNS, staVNS), which is stimulation of the earlobe with the same parameters as taVNS (participants do not know the distribution of VN in the earlobe) and the taVNS device is not switched off. A summary of ear stimulation targets is shown in Fig 1.

In study 2, we created four different combinations of stimulation parameters based on pulse width (50 us, 250 us) and frequency (5 Hz, 20 Hz) in order to cover a wide range (low to high) of both pulse width and frequency. Subjects were not required to attend for a sham visit (rationale based on study 1 results) and received only taVNS with different parameters. The stimulation intensity was unified and adjusted to perceptual threshold (PT) level.

### ECG analysis

After the ECG signal recorded, we used MATLAB R2016a to process it for extraction of tachogram, which contains the inter-beat-intervals (IBIS) which was required for HRV analyzing. Firstly, we employed the Pan-Tompkins algorithm to detect the R peaks of the ECG signal [26]. Secondly, we visually inspected the detection and manually removed artifacts according to published guidelines [27]. Finally, we extracted time- and frequency-domain indices of HRV from the processed IBIs. Based on the recommendations for HRV measurement [27], we analyzed time domain and frequency domain indicators of HRV with HRV analysis software (version 1.0) in blocks of 5 min.

In HRV analysis, several parameters in frequency-domain and time-domain were mainly focused. In time-domain parameters, RMSSD and pRR50 are associated with parasympathetic output, SDRR can reflect the overall variability in heart rate (HR) [27]. Frequency-domain HRV parameters include: total power (TP), detected at 0.04–0.40Hz, the low frequency (LF) component, detected at 0.04–0.15Hz, can reflect both sympathetic and parasympathetic modulation of heart rate, while the high frequency (HF) component, which detected at 0.15–0.40Hz, is associated with parasympathetic modulation of heart rate [27], the ratio of LF to HF power can be used as an index of the balance of sympathetic and parasympathetic activity [28].

### Procedure

All studies were carried out from 09:00 a.m. to 12:00 a.m. in a quiet laboratory at the temperature of 26±2°C. Participants were required to sit quietly, stay awake and remain silent except reporting subjective response (pain ratings or tension ratings). During the period of visit, they wear ear electrode clips. To avoid cardiac side effects, all electrical stimulations were performed at the left ear [29].

#### Study 1 procedure

**Fig 2:**
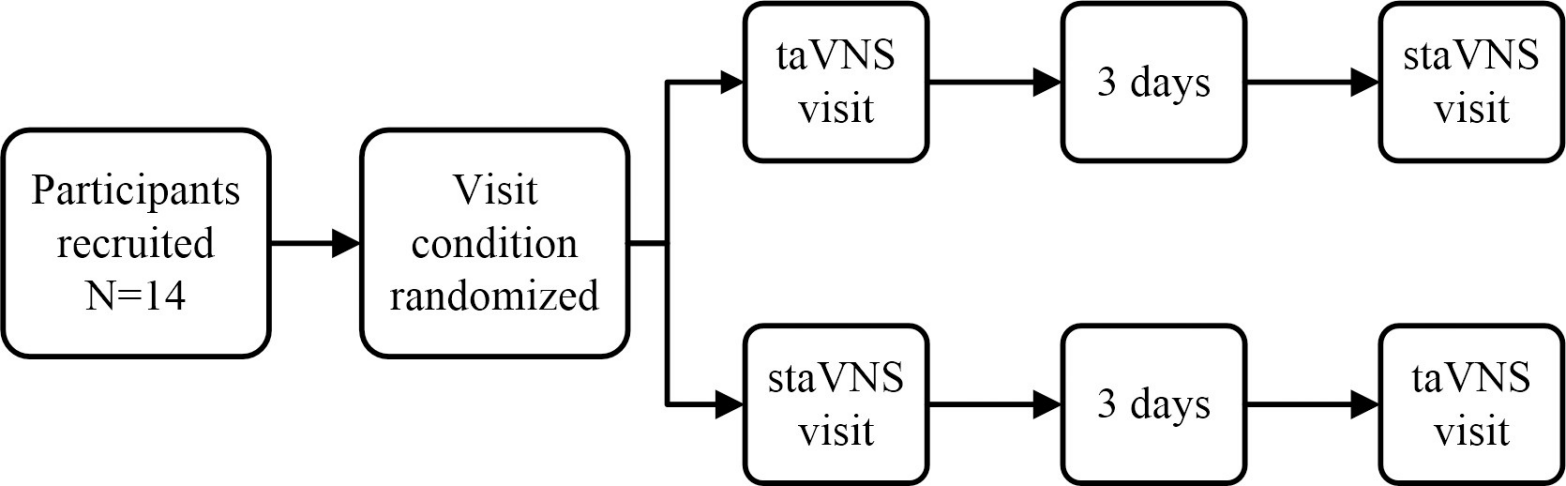
Procedure for study 1. Fourteen participants were randomly assigned to receive either taVNS or staVNS at the beginning and then another opposite stimulation three days later. Each round consisted of three phases: 5min baseline, 5min stimulation, 5min recovery.

In study 1, we performed a two-stage cross-over trial, investigating the difference of effects on HRV between taVNS and staVNS conditions. 14 participants were enrolled in this study and required to complete a short questionnaire, including the background information of age, gender, height, weight, and physical activity before the experiment. After the ear electrode clips, the physiological equipment for continuous recording of ECG and respiratory data connected, all participants rested for 10 minutes before the visit beginning. Each visit consisted of three blocks: 5-minute baseline, 5-minute stimulation and 5-minute recovery. Fig 2 summarizes the detailed procedures for this study.

#### Study 2 procedure

**Fig 3:**
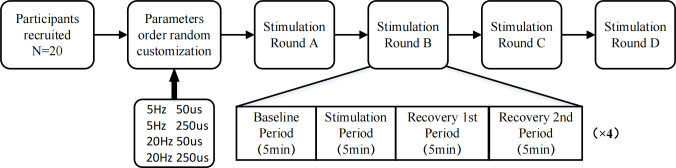
Procedure for study 2. Twenty participants were recruited. Each of them was subjected to taVNS with four different parameters. The order of the parameters was randomly customized. Each round of stimulation consisted of four phases: baseline, stimulation, recovery1st and rcovery2nd.

Study 2 explored whether taVNS has parameter-specific effects on HRV. The program of this study was similar wtih that of Study 1, but Study 2 did not require staVNS. 20 participants were enrolled in this study, and each of them received four rounds of taVNS with different parameters (one round per parameter). Each round consisted of four sub-blocks: baseline, stimulation, recovery 1st and recovery 2nd, and there was the 5-min time length in each sub-block. In order to eliminate out order effects, the order of stimulations was randomly tailored for each participant. Fig 3 summarizes the detailed procedures for this study.

### Measurements

The electrocardiogram (ECG) was measured using a three-lead ECG acquisition equipment (PC-80B, Shanghai Lixin Instrument Co, Ltd.) in order to assess HRV. ECG data was recorded using three AgCl surface electrodes, on both sides of the 4th intercostal space of the sternum and one left electrode on the costal margin of the right midclavicular line. According to the suggestion of previous studies [30], the sampling rate was 1024Hz. Abdominal respiration was measured by using a flexible belt (PC-80B, Shanghai Lixin Instruments Co, Ltd.) at a sampling rate of 32 Hz in order to record respiratory data and exclude the participants with abnormal respiratory rate (minimum respiration rate of ≤10 breaths per minute).

In both studies, participants were required to complete a short questionnaire to exclude the people who did not meet the criteria, including background information of age, gender, height, weight, and physical activity before the experiment. Stimulation intensity during each visit was recorded by experimenters. In addition, after each of stimulation period, participants were instructed to report subjective responses using a Likert scale ranging from 0 to 9 that were explicitly related to the past stimulation period. “0” was used as the lowest rating for no sensation perceived or feel no tension; “9” was the highest pain rating for extreme or feel very tension [31].

### Statistical analysis

All data were presented as the mean ±0.01 standard deviation (SD) unless specially stated and statistically analyzed using SPSS (version 22). Shapiro-Wilk was conducted to test the normality of the distribution. Two-tailed statistical tests were used in all instances with an alpha level of 0.05.

#### Study 1

For subjective ratings (pain ratings, tension ratings) and stimulation intensity, paired samples t-tests were performed to explore differences between the taVNS and staVNS with the condition (taVNS and staVNS) as a normally distributed within-subject factor. For non-normally distributed data, wilcoxon signed-rank tests examined the difference between taVNS and staVNS in respiration. One-way repeated measures ANOVA examined the differences in tension ratings between different stages of visit (baseline, stimulation, recovery). To explore which stimulation conditions (taVNS or staVNS) caused a greater increase in HRV parameters, two-way repeated measure ANOVAs were performed. Greenhouse-geisser was used for correction when the data did not meet the spherical hypothesis test. If there were significant main effects or interaction effects, post hoc t-tests with Bonferroni correction were calculated for pairwise comparisons. Linear regressions examined whether baseline measures of HRV indexes significantly predicted response (change (Δ) between baseline and stimulation).

#### Study 2

To assess the differences between four stimulation conditions in stimulation intensity, pain ratings, tension ratings and respiration, Friedman’s test was performed with stimulation condition as a nonnormally distributed within-subject factor. One-way repeated measures ANOVA was performed to assess the residual effect of taVNS from the previous stimulation round to the next one. To explore whether taVNS has parameter-specific effects, two-way repeated measure ANOVAs with Bonferroni correction was conducted, investigating the differences in the effects of taVNS on HF power between different stimulation parameters. In addition, Spearman correlations examined the correlation between measurement of HF power and stimulation intensity.

## Results

### Study 1

Fourteen young participants (8 males, 6 females, mean age: 23.42±1.29 years, mean BMI:22.3±1.32) were enrolled in study 1, no abnormalities were observed in HR (less than 60 beats or more than 100 beats per minute) or respiration during the visit, and no adverse effects were reported after the trial ended.

#### Stimulation intensity and subjective responses

Paired sample t-tests revealed that there was no significant difference in the stimulation intensity between taVNS and staVNS (taVNS: 14.79±6.02mA, staVNS: 13.91±5.28mA, p = 0.44). Similarly, there was no significant difference in pain ratings between taVNS and staVNS (taVNS: 2.18±1.36; staVNS: 1.82±0.97, p = 0.46). According to wilcoxon signed-rank tests, there was no significant difference in respiration rate between taVNS and staVNS (taVNS: Mdn = 15; staVNS: Mdn = 16, z = -1.389, p = 0.165).

When the subjective tension ratings were investigated, one-way repeated measure ANOVAs showed that the ratings of tension during the stimulation were significantly higher than those at baseline under both taVNS and staVNS groups (taVNS, p = 0.0013; staVNS, p = 0.002). However, paired sample t-test showed that there were no significant differences between taVNS and staVNS during the stimulation (p = 0.812), see Table 1.

**Table 1:** Tension ratings at different period under taVNS or staVNS.

Condition	Baseline	Stimulation	Recovery	p-value
taVNS	0.78±0.55	3.07±0.79*	1.28±0.45	0.017
staVNS	0.85±0.36	2.78±0.77*	0.92±0.71	0.029

Statistically significant differences between the three recordings (baseline, stimulation, recovery) transpired. Reported p-values are from the one-way ANOVAs. * = significantly different to baseline (post-hoc tests). Data presented as the mean ± SD.

#### taVNS vs. staVNS effects on HRV measures

**Fig 4:**
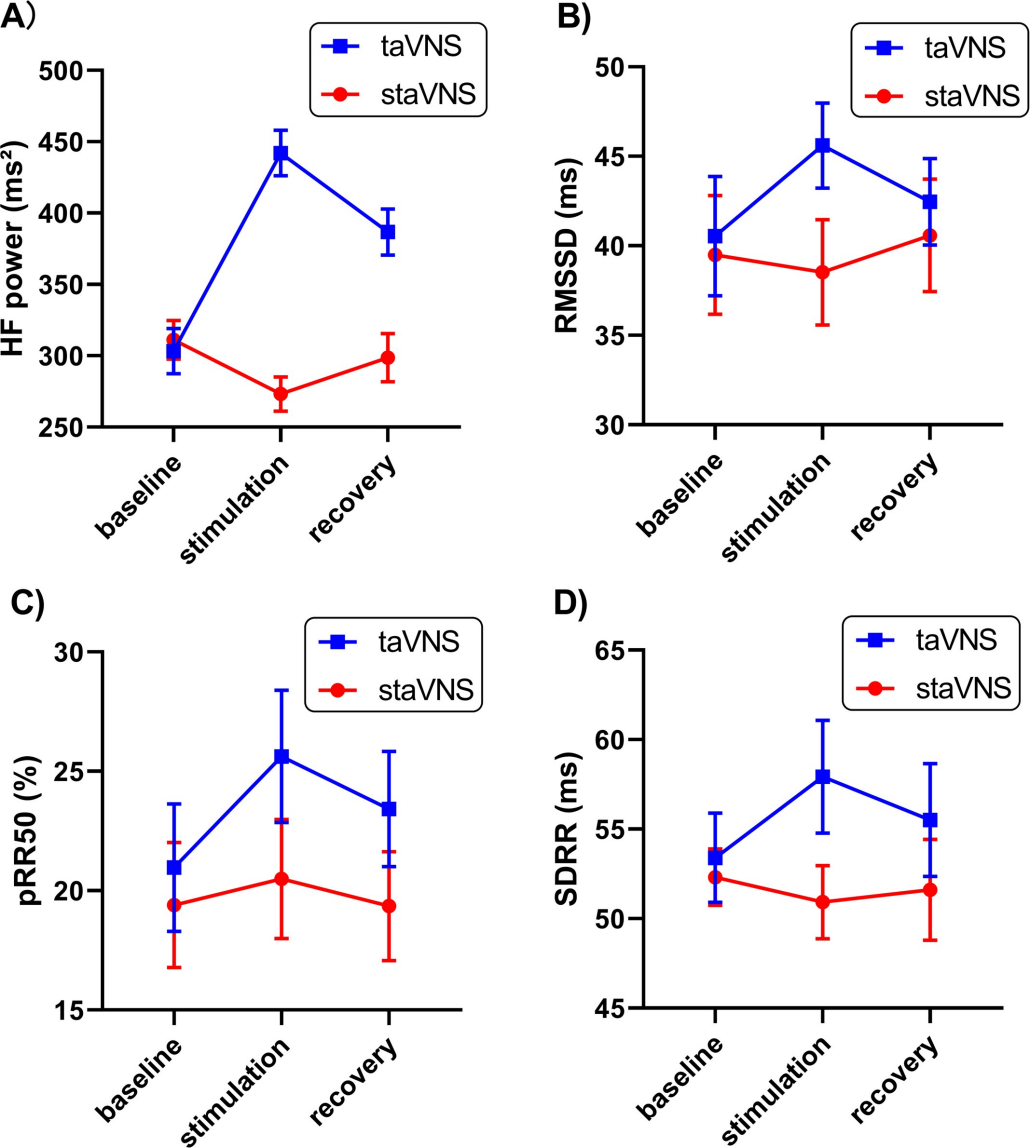
The response of autonomic function measured by HRV in taVNS or staVNS conditions over time: (A) HF, (B) RMSSD, (C) pRR50, (D) SDRR. In taVNS, the measurements of HF, RMSSD, PRR50 and SDRR were significantly higher than those in staVNS.

Descriptive statistics of HRV indicators over time under taVNS or staVNS conditions are presented in Table 2 and the results of two-way repeated measure ANOVAs for various HRV indicators can be found in Table 3. Two-way repeated measure ANOVAs showed that measures of cardiac vagal activity: HF power (Fig 4A), RMSSD (Fig 4B), and pRR50 (Fig 4C) were significantly higher under the taVNS than the staVNS conditions (main effect of condition: HF power, F (1,13) = 9.732, p = 0.001, η_p_^2^ = 0.178; RMSSD, F (0.901,11.635) = 3.928, p = 0.026, η_p_^2^ = 0.082; pRR50, F (1,13) = 3.816; p = 0.027, η_p_^2^ = 0.139). Besides, measures of SDRR (Fig 4D), which reflecting overall HRV, were significantly higher under the taVNS than the staVNS conditions (main effect of condition: SDRR, F (1,13) = 3.629, p = 0.037, η_p_^2^ = 0.025).

**Table 2:** Descriptive statistics of HRV indicators among the three recordings (baseline, stimulation, recovery) under taVNS or staVNS conditions.

	taVNS			staVNS		
HRV index	Baseline	Stimulation	Recovery	Baseline	Stimulation	Recovery
RMSSD	40.5±2.9	45.6±2.1	42.5±2.5	39.5±2.7	38.5±2.6	40.6±2.8
pRR50	20.9±2.4	25.6±2.5	23.4±2.2	19.4±2.3	20.5±2.2	19.4±2.1
SDRR	53.4±2.2	57.9±2.8	55.5±2.8	52.3±1.4	50.9±1.8	51.6±2.5
HF	303.2±14.1	442.0±14.3	386.6±14.1	311.1±12.1	273.1±10.7	298.5±15.1
LF	661.1±380.8	799.0±287.2	742.4±315.4	621.3±330.2	557.2±396.8	677.7±310.9
LF/HF	2.1±1.1	1.8±1.0	1.9±0.8	2.0±0.9	2.1±1.0	2.3±1.2
TP	2216.7±875.1	2784.7±293.2	2364.6±647.6	2204.2±1581.8	1748.1±847.5	1964.8±742.5

Data presented as the mean ± SD.

**Table 3:** Results of two-way repeated measure ANOVAs for various HRV indicators.

HRV index	main or interaction effect	*F*-value	*p*-value	η_p_^2^
RMSSD	Time measurements	1.76	0.2	n.s.
	Stimulation condition	3.92	0.03*	0.726
	Time × condition	5.94	0.02*	0.082
pRR50	Time measurements	3.75	0.03*	0.012
	Stimulation condition	3.81	0.03*	0.139
	Time × condition	1.71	0.26	n.s.
SDRR	Time measurements	0.98	0.57	n.s.
	Stimulation condition	3.62	0.04*	0.025
	Time × condition	1.28	0.46	n.s.
HF	Time measurements	2.91	0.13	n.s.
	Stimulation condition	9.73	0.001*	0.178
	Time × condition	5.79	0.02*	0.017
LF	Time measurements	0.41	0.88	n.s.
	Stimulation condition	1.12	0.49	n.s.
	Time × condition	1.01	0.58	n.s.
LF/HF	Time measurements	2.59	0.08	n.s.
	Stimulation condition	2.84	0.07	n.s.
	Time × condition	1.37	0.26	n.s.
TP	Time measurements	2.38	0.22	n.s.
	Stimulation condition	0.97	0.46	n.s.
	Time × condition	1.09	0.1	n.s.

Reported p-values are from the two-way ANOVAs.

* = significantly main effect. Data presented as the mean ± SD.

Two-way repeated measure ANOVAs also showed a main effect of measurement time for pRR50 (F (1.662, 24.623) = 3.758, p = 0.031, η_p_^2^ = 0.012). Post hoc analysis of three pairwise comparisons, with Bonferroni correction (corrected p = 0.017), revealed that the measurement of pRR50 was significantly higher during the stimulation compared to the baseline (p = 0.002, d = 0.441), but no significant differences between recovery vs baseline (p = 0.262) or recovery vs stimulation (p = 0.386).

#### taVNS’s carry-over effect

**Fig 5:**
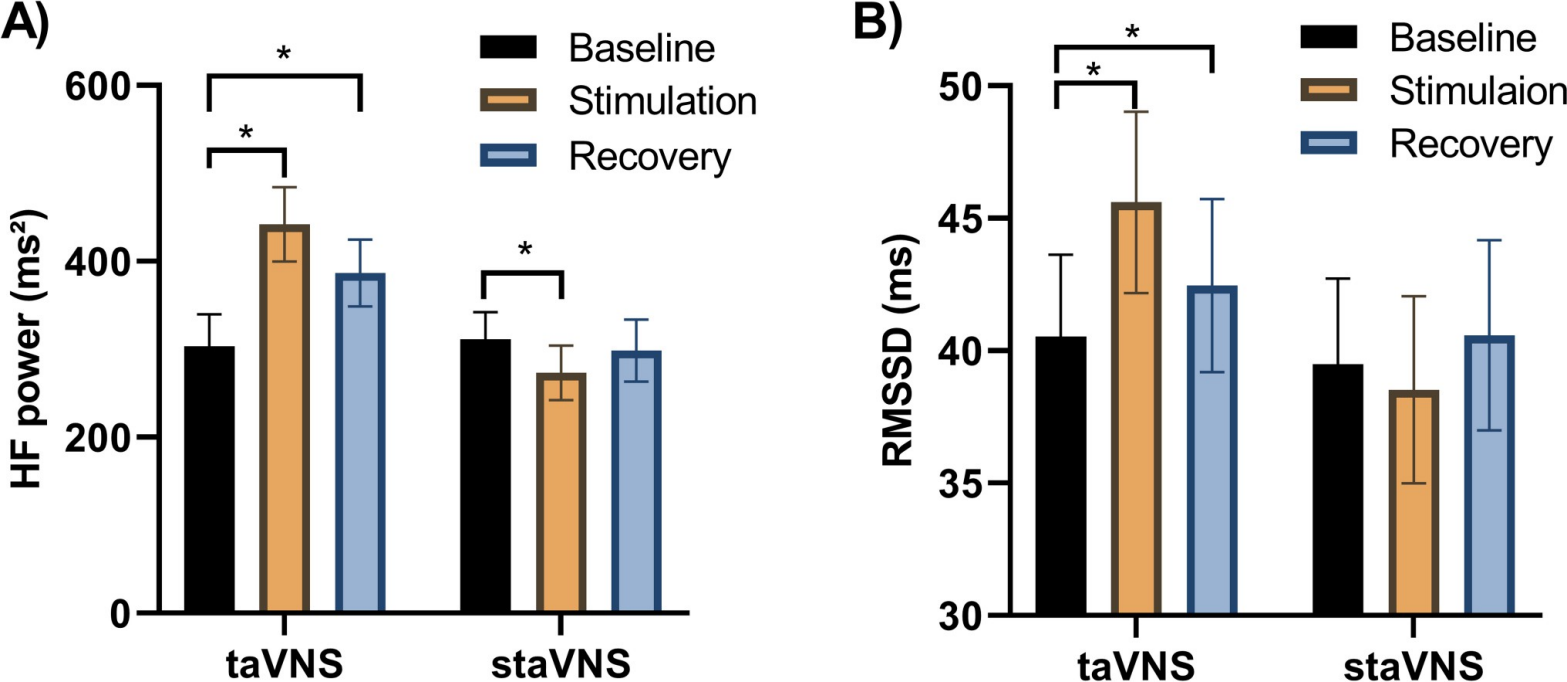
Time course analysis for HF (A) and RMSSD (B) by the taVNS or staVNS conditions. Measurements of vagal tone: RMSSD and HF significantly increased from baseline to stimulation and recovery period in taVNS condition, whereas HF significantly decreased from baseline to stimulation period in staVNS condition. Post hoc simple effect analysis of time with Bonferroni corrected, *p<0.0017.

The study showed significant interaction effect of time × condition for measures of vagal tone: HF power and RMSSD (F (1.889,25.314) = 5.794, p = 0.019, η_p_^2^ = 0.017; F (1.476,22.179) = 5.942, p = 0.017, η_p_^2^ = 0.082, respectively). Further analysis revealed that both measurements of RMSSD and HF power were significantly higher during stimulation period than baseline period in taVNS condition (RMSSD, p = 0.0017, d = 0.497; HF power, p = 0.0001, d = 0.561). In addition, the study also found a significant increase from baseline to recovery period in both RMSSD and HF power (RMSSD, p = 0.011, d = 0.269; HF power, p = 0.006, d = 0.401, see Fig 5).

What should be pointed out is that the measurement of HF power was significantly decreased from baseline period to stimulation period in staVNS condition (p = 0.014, d = 0.211).

#### Baseline HRV can predict response to taVNS

**Fig 6:**
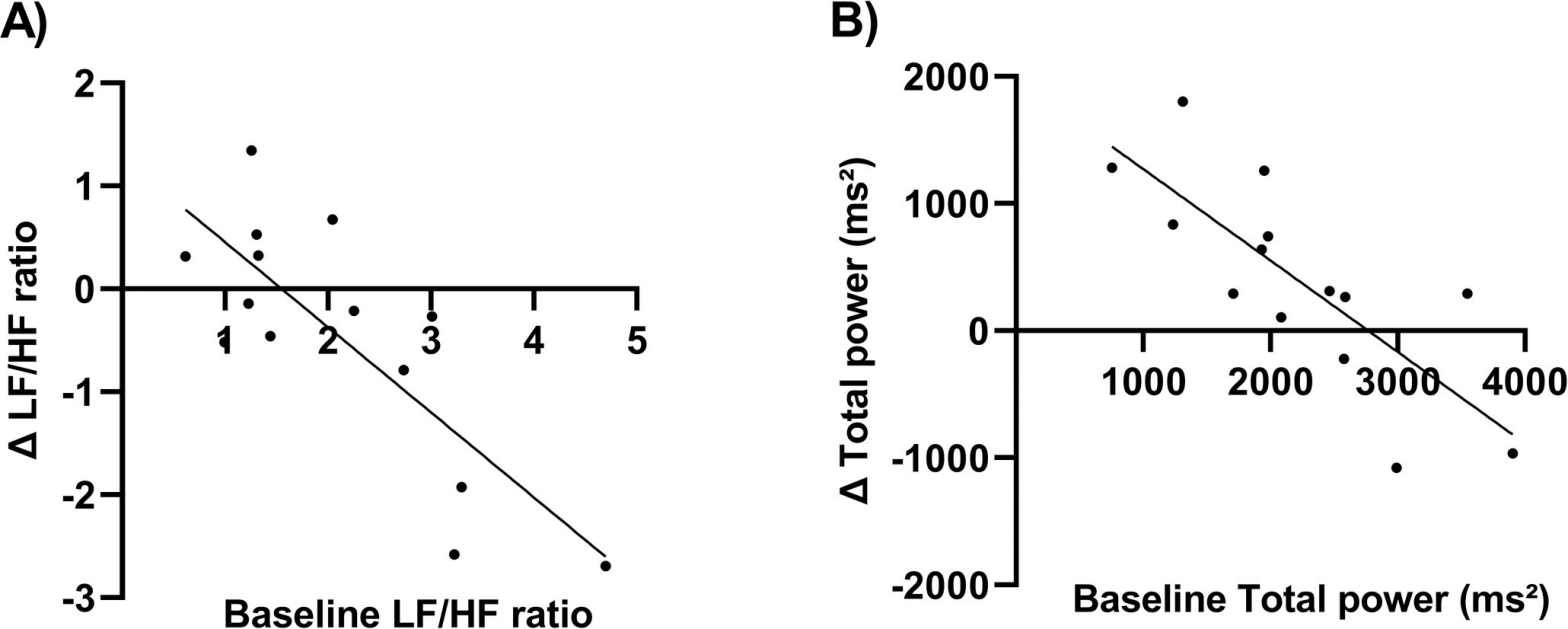
The baseline LF/HF ratio and TP can predict response to taVNS: (A) Higher baseline LF/HF ratio was associated with greater LF/HF ratio decreases (R^2^ = 0.691, p = 0.0001), (B) lower baseline TP was associated with greater increases in TP (R ^2^ = 0.459, p = 0.004).

We examined the relationship between baseline HRV indices and the change of it after taVNS. A linear regression analysis revealed an association between higher baseline LF / HF and greater LF / HF decreases (R^2^ = 0.691, p = 0.0001, Fig 6A). In addition, it is found that there was also a linear relationship between TP and Δ TP, where lower baseline TP was associated with greater increases in TP during taVNS (R ^2^ = 0.459, p = 0.004, Fig 6B)

### Study 2

Another twenty young participants (11 males, 9 females, mean age: 21.51±2.12 years, mean BMI:21.6±1.47) were enrolled in the study, no abnormalities were observed in HR or respiration during the visit, and no adverse effects were reported after the trial ended.

#### Stimulation intensity and subjective responses

**Fig 7:**
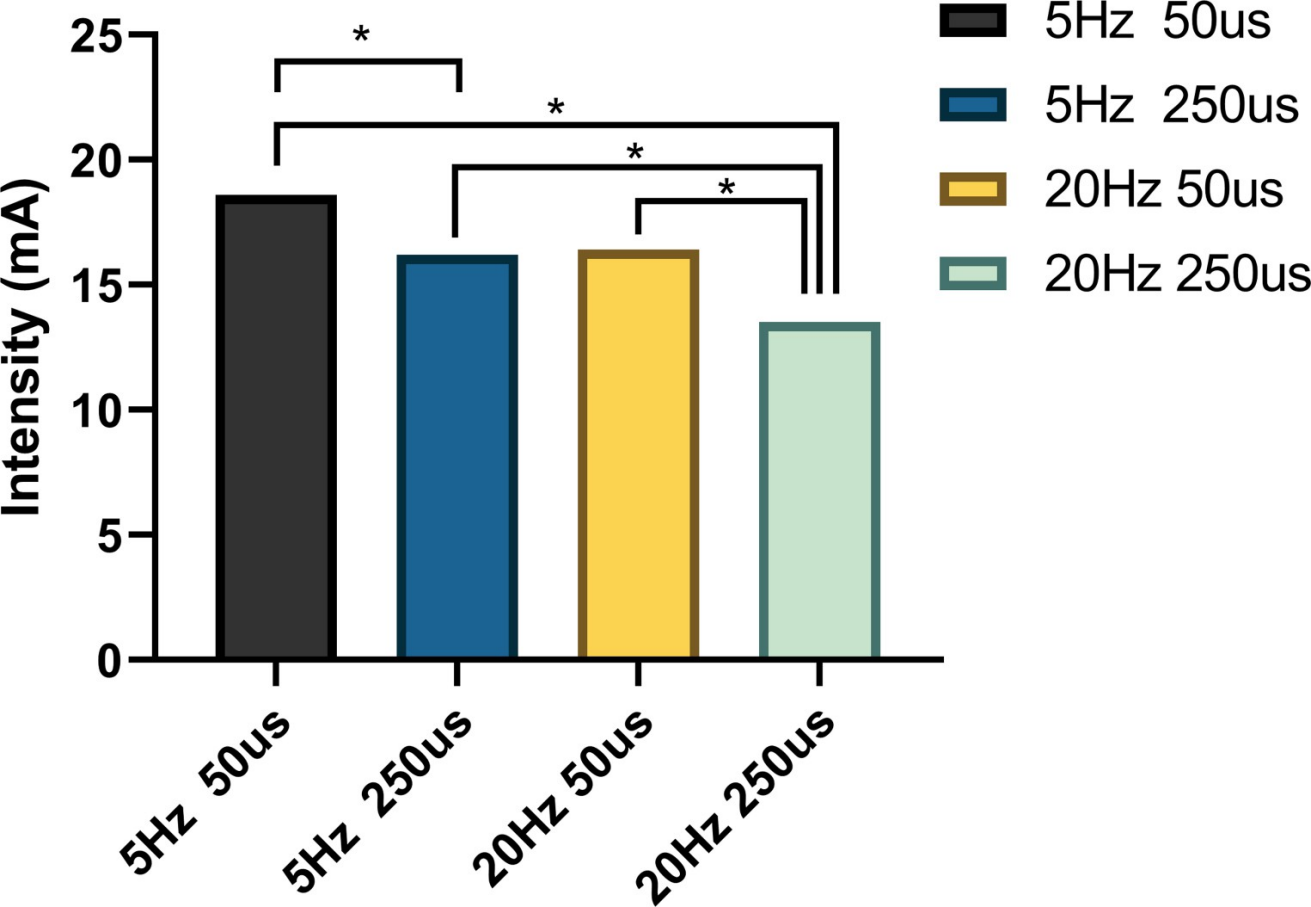
Differences in stimulation intensity between various parameters. The 20Hz 250us parameter was significant weaker than the 5Hz 50us parameter (p = 0.0001), the 5Hz 250us parameter (p = 0.0017), as well as the 20Hz 50us parameter (p = 0.0009). In addition, the 5Hz 250us parameter was significant weaker than the 5Hz 50us parameter (p = 0002). Statistical analysis by Friedman test with bonferroni corrected, *p<0.0083.

Descriptive statistics of Stimulation intensity, pain ratings and tension ratings under different stimulation conditions can be seen in Table 4. As for the stimulation intensity, a Friedman test revealed that stimulation intensities during various stimulation conditions were significantly different (χ^2^ (3) = 16.879, p = 0.0012). A post hoc analysis of six pairwise comparisons (Bonferroni-corrected, p = 0.0083) showed that the 20Hz 250us parameter (Mdn = 13.5) was significantly weaker than the 5Hz 50us parameter (Mdn = 18.5, p = 0.0001), and the 5Hz 250us parameter (Mdn = 16.0, p = 0.0017), as well as the 20Hz 50us parameter (Mdn = 16.5, p = 0.0009). In addition, the 5Hz 250us parameter was significant weaker than the 5Hz 50us parameter, p = 0002. See Fig 7.

**Table 4:** Descriptive statistics of Stimulation intensity, pain ratings and tension ratings under different stimulation conditions.

	5Hz 50us	5Hz 250us	20Hz 50us	20Hz 250us
Stimulation intensity	18.85±3.87	16.45±3.36	16.70±3.71	13.55±2.78
Pain ratings	2.11±1.01	1.97±0.79	2.06±1.24	1.85±1.13
Tension ratings	3.21±1.07	2.98±0.82	3.44±1.26	2.83±0.99
Respiration rates	15.14±1.41	15.92±1.48	15.42±1.39	15.85±1.81

As to pain ratings, a Friedman test showed that there was no significant difference between various stimulation conditions (χ^2^ (3) = 2.641, p = 0.053).

In a similar vein, regarding subjective tension ratings, it was shown that participants’ tension ratings not differed significantly during various stimulation conditions (χ^2^ (3) = 1.584, p = 0.062). There was no significant difference in the respiration rate between various stimulation conditions (χ^2^ (3) = 1.139, p = 0. 67).

#### Estimation of residual effects on HF power

In order to estimate the residual effect of taVNS in the previous stimulation round to the next one, one-way repeated measures ANOVA was performed, checking the difference of HF power measurements in the baseline period between different stimulation rounds. The result pointed out that there was no significant difference in HF power measurements between baseline periods of different stimulation rounds (p = 0.512).

#### Comparison of taVNS effects between different parameters

**Fig 8:**
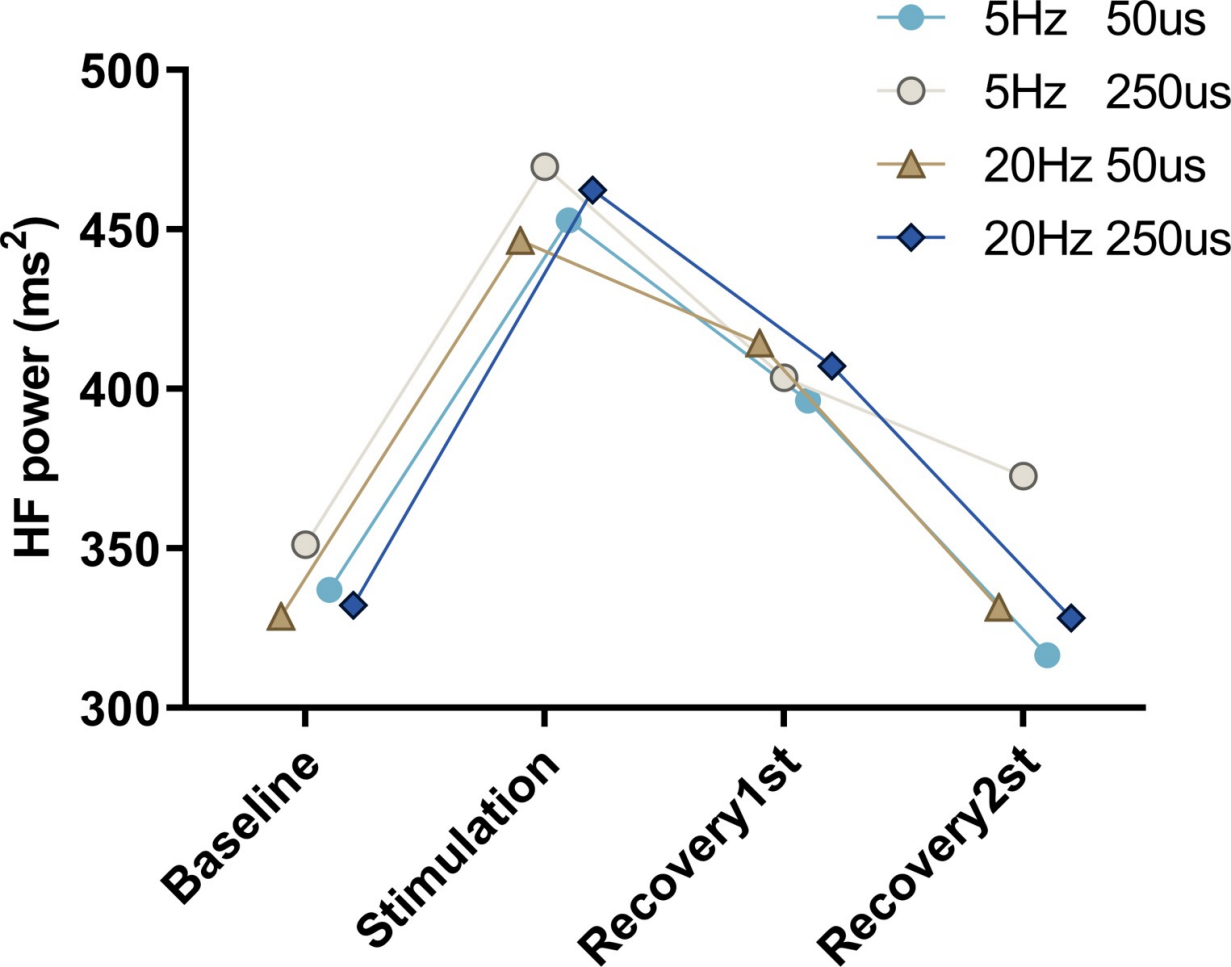
Stimulation response by parameter over time. TaVNS significantly increased the measurement of HF during stimulation and recovery 1st than baseline period, but there was not significantly different between various parameters. Statistical analysis by two-way repeated measures ANOVA.

A repeated measures ANOVA showed a main effect of measurement time for HF power when values among four stimulation conditions were combined across each round (F (2.616,47.961) = 6.982, p = 0.009, η_p_^2^ = 0.078). Post hoc analysis of six pairwise comparisons, with Bonferroni correction (corrected p = 0.0083), revealed a significant increase from baseline to stimulation (p = 0.002, d = 0.339), and the change persisted into the first half of the recovery (p = 0.007, d = 0.352) rather than the second half of recovery (p = 0.87). In addition, we found a significant decrease from stimulation to the second half of recovery (p = 0.0027, d = 0.413), and no significant difference in HF power between baseline and the second half of recovery (p = 0.73). No significant main effect of stimulation condition, F (3, 57) = 1.332, p = 0.48, or interaction effect of time×condition, F (7.387,136.854) = 1.016, p = 0.56, for HF power was found (Fig 8).

Considering stimulation intensity varied significantly under different stimulation conditions, spearman tests were performed, which showed no correlation between stimulation intensity and measurement of HF power (rs = 0.102, p = 0.77)

## Discussion

This study showed that compared with staVNS, taVNS could change HRV of healthy young people, significantly improving the measurements of RMSSD, SDRR, pRR50 and HF power. Significantly, the effect of taVNS was not confined to the stimulation period. On the contrary, it had a carry-over effect. Intriguingly, the measurement of LF/HF ratio in baseline period could significantly predict the response from participants to taVNS. In addition, findings showed no significant difference in effect of different parameters of taVNS on HF power.

### The effects of taVNS on HRV

Some studies have investigated the effects of vagus nerve stimulation on HRV, but the results were mixed. For example, De Couck found taVNS significantly increased SDNN compared to baseline without the effects on RMSSD, HF or LF/HF [32]. Contrastively, two other studies indicated that taVNS significantly decreased LF/HF ratio without the significant effects on other indexes of HRV [33, 34]. Significantly, their studies compared active stimulation to a “stimulation off” sham condition [32, 33] or a pre-stimulation baseline [34]. Considering that pain sensation or tension emotion caused by taVNS as well as respiration (could be increased by tension) would affect HRV [35–37], which might cause irrelevant variables and affect the interpretation of the results. We therefore stimulated the earlobe with the same parameters as taVNS as sham control condition. The earlobe is thought to be relatively free of vagal afferents [25]. Besides, the present study showed no significant difference in pain ratings, tension ratings or respiration rates between taVNS and staVNS. Therefore, it is thought that the potential influence of the irrelevant variables on the results was eliminated and the comparability between the active and control was increased.

The two-way repeated measurement ANOVAs analyses conducted in study 1 demonstrated that taVNS significantly increased the measurements of RMSSD、pRR50、HF、SDRR. The conclusion differed from previous findings, and some further possible explanations for these differences in the results can be found out. Firstly, different types of “control” conditions were used in this study and previous studies. Secondly, the present study and most previous studies have used relatively small sample sizes, meaning that it may have been statistically underpowered to detect potentially meaningful effects as significantly different from zero. Thirdly, stimulation parameters vary considerably between studies which may diminish the comparability between studies. Finally, there is a large heterogeneity between studies in the tested samples, ranging from healthy young adults to the elderly to heart failure patients.

This study provides the evidence that taVNS could increase cardiac vagal activity. However, as the auricular branch of the vagus nerve (ABVN) only consists of afferent fibers, effects of taVNS on HRV are necessarily indirect. Specifically, as proposed by Murray and colleagues [38], taVNS may increase input to the nucleus tractus solitarii (NTS), thereby increasing the activity of NTS neurons projecting to the two vagal efferent nuclei: the dorsal motor nucleus and the nucleus ambiguus. Increased activation in these nuclei may, in turn, increase vagal control of cardiac activity.

It is particularly interesting that the effect of taVNS had a carry-over effect on HRV. It is found that the measurements of vagal tone (HF, RMSSD) were still significantly higher during recovery period than in baseline period. A similar pattern of results was obtained in previous studies, for example, HF power could increase at least an hour after finishing taVNS by using acupuncture [39]. Clancy and colleagues found that the LF/HF ratio and muscle sympathetic nerve activity (MSNA) remained lower than baseline levels during the recovery period after taVNS had ceased [33]. Besides, a previous functional magnetic resonance imaging (fMRI) finding also showed that the nucleus tractus solitarius was continuously activated for 11 minutes after the cessation of taVNS [40]. Therefore, the long-term effect of taVNS on VN requires further investigation, which is of great significance for the application of taVNS. Future studies should take the measurement of VN activity as the dependent variable, and systematically investigate the carry-over effect of taVNS with different duration. The finding may provide some reference for subsequent taVNS studies. Considering that there is a persisting effect on HRV, if two or more taVNS are to be administered in a short period of time, a sufficiently large washout period should be set to estimate the residual effect of taVNS in the previous stimulation round to the next one. Finally, whether the duration of taVNS’ effect on HRV is related to the duration of taVNS needs to be systematically tested in subsequent studies.

Baseline LF/HF ratio, a measure of autonomic balance, was a significant predictor of participants’ response to taVNS: higher baseline LF/HF ratio was associated with greater LF/HF ratio decrease. It implied that humans with higher sympathetic activity are subjected to a stronger taVNS effect. These patterns emerged in previous work and may potentially contribute to screen individuals who are likely to encounter greater autonomic benefits from taVNS [33]. Such a prediction is particularly important considering that there are more than 100 diseases associated with reduced VN activity, which are widely distributed in cardiovascular, endocrine and metabolic systems [41]. In addition, this would help to develop more effective inclusion criteria in order to carry out more targeted studies for taVNS.

### taVNS’s effects of parameter

There have been several studies exploring the optimal parameters for taVNS. For example, a fMRI study provided evidence that the optimum stimulation intensity is 8 mA without perception of pain, which caused the maximum amplitude of vagus somatosensory evoked potentials [42], another parametric study found that higher pulse width and stimulation frequency (i.e., 200 and 500μs pulse width, 10 and 25Hz) were associated with more pronounced cardiac deceleration [17]. Nevertheless, these studies did not investigate the effect of different parameters of taVNS on HRV. This study hypothesized that parameters of higher energy density (larger pulse width, higher frequency) would be more effective in enhancing of HF power measurement. In fact, there is an infinite combination of frequency, pulse width, and duty cycle that would be impossible to exhaustively test. Limited by experimental conditions, the second trial conducted therefore was not an exhaustive exploration of parameters but rather a reasonable combination of high and low settings based on prior optimal parameters study. However, we found no significant difference in the effect of taVNS on the measurement of HF power between different parameters. This may be due to the smaller sample size used (n = 20), the large range of parameters which makes it impossible to exhaustively test.

Significantly, stimulation intensity showed a significant difference between different parameters, the parameters with lower energy density appeared to require more stimulation intensity than the higher. However, spearman’s test showed no significant correlation between stimulation intensity and HF measurement. One possible explanation is that there may be a maximum effect threshold of taVNS, i.e., when the stimulation intensity reaches a certain degree, its effect is no longer enhanced with increasing intensity. It is possible that the maximum effect threshold was already exceeded when stimulation intensities reached perceptual threshold levels, so effects on HF power did not differ despite differences in stimulation intensities. Further studies should consider this assumption and set different stimulation intensities for taVNS in a large range, comparing the effects of different stimulation intensities on HF power or other HRV index.

### Limitations

The main limitation of our study was that the study was based on a sample of healthy young adults, perhaps a larger taVNS effect could be observed in the elderly or in some patients where cardiovascular autonomic balance is shifted toward sympathetic predominance such as heart failure, hypertension, which would require further testing. Besides, HRV represents a biomarker for efferent vagal activation, whereas many research on taVNS focuses on afferent effects of on cognitive, emotional, or neurological functioning. The effect of taVNS on HRV does not necessarily indicate an effect on intracranial structures. Possible additional markers could be salivary alpha amylase, pupillary responses or P300 for norepinephrine release [43, 44].

## References

[1] Groves DA, Brown VJ. Vagal nerve stimulation: A review of its applications and potential mechanisms that mediate its clinical effects. Neurosci Biobehav Rev. 2005;29: 493–500. doi: 10.1016/j.neubiorev.2005.01.004 15820552

[2] Berthoud H-R, Neuhuber WL. Functional and chemical anatomy of the afferent vagal system. Auton Neurosci Basic Clin. 2000;85: 1–17. doi: 10.1016/S1566-0702(00)00215-0 11189015

[3] Thayer JF, Yamamoto SS, Brosschot JF. The relationship of autonomic imbalance, heart rate variability and cardiovascular disease risk factors. Int J Cardiol. 2010;141: 122–131. doi: 10.1016/j.ijcard.2009.09.543 19910061

[4] Zulfiqar U, Jurivich DA, Gao W, Singer DH. Relation of High Heart Rate Variability to Healthy Longevity. Am J Cardiol. 2010;105: 1181–1185. doi: 10.1016/j.amjcard.2009.12.022 20381674

[5] de Lartigue G. Role of the vagus nerve in the development and treatment of diet‐induced obesity. J Physiol. 2016;594: 5791–5815. doi: 10.1113/JP271538 26959077 PMC5063945

[6] George MS, Sackeim HA, Rush AJ, Marangell LB, Nahas Z, Husain MM, et al. Vagus nerve stimulation: a new tool for brain research and therapy*. Biol Psychiatry. 2000;47: 287–295. doi: 10.1016/s0006-3223(99)00308-x 10686263

[7] Shahwan A, Bailey C, Maxiner W, Harvey AS. Vagus nerve stimulation for refractory epilepsy in children: More to VNS than seizure frequency reduction. Epilepsia. 2009;50: 1220–1228. doi: 10.1111/j.1528-1167.2008.01940.x 19170732

[8] Milby AH, Halpern CH, Baltuch GH. Vagus nerve stimulation for epilepsy and depression. Neurotherapeutics. 2008;5: 75–85. doi: 10.1016/j.nurt.2007.10.071 18164486 PMC5084129

[9] De Ferrari GM, Crijns HJGM, Borggrefe M, Milasinovic G, Smid J, Zabel M, et al. Chronic vagus nerve stimulation: A new and promising therapeutic approach for chronic heart failure. Eur Heart J. 2011;32: 847–855. doi: 10.1093/eurheartj/ehq391 21030409

[10] Val-Laillet D, Biraben A, Randuineau G, Malbert CH. Chronic vagus nerve stimulation decreased weight gain, food consumption and sweet craving in adult obese minipigs. Appetite. 2010;55: 245–252. doi: 10.1016/j.appet.2010.06.008 20600417

[11] Ness TJ, Randich A, Fillingim R, Faught RE, Backensto EM, Birklein F, et al. Left vagus nerve stimulation suppresses experimentally induced pain [3] (multiple letters). Neurology. 2001;56: 985–986. doi: 10.1212/wnl.56.7.985 11294950

[12] Elliott RE, Morsi A, Kalhorn SP, Marcus J, Sellin J, Kang M, et al. Vagus nerve stimulation in 436 consecutive patients with treatment-resistant epilepsy: Long-term outcomes and predictors of response. Epilepsy Behav. 2011;20: 57–63. doi: 10.1016/j.yebeh.2010.10.017 21144802

[13] Fang J, Rong P, Hong Y, Fan Y, Liu J, Wang H, et al. Transcutaneous vagus nerve stimulation modulates default mode network in major depressive disorder. Biol Psychiatry. 2016;79: 266–273. doi: 10.1016/j.biopsych.2015.03.025 25963932 PMC4838995

[14] Rong P, Liu A, Zhang J, Wang Y, He W, Yang A, et al. Transcutaneous vagus nerve stimulation for refractory epilepsy: a randomized controlled trial. Clin Sci. 2014. doi: 10.1042/CS20130518 24684603

[15] Kaniusas E, Kampusch S, Tittgemeyer M, Panetsos F, Gines RF, Papa M, et al. Current directions in the auricular vagus nerve stimulation I–A physiological perspective. Front Neurosci. 2019;13: 1–23. doi: 10.3389/fnins.2019.00001 31447643 PMC6697069

[16] Kaniusas E, Szeles JC, Kampusch S, Alfageme-Lopez N, Yucuma-Conde D, Li X, et al. Non-invasive Auricular Vagus Nerve Stimulation as a Potential Treatment for Covid19-Originated Acute Respiratory Distress Syndrome. Front Physiol. 2020;11: 890. doi: 10.3389/fphys.2020.00890 32848845 PMC7399203

[17] Badran BW, Mithoefer OJ, Summer CE, LaBate NT, Glusman CE, Badran AW, et al. Short trains of transcutaneous auricular vagus nerve stimulation (taVNS) have parameter-specific effects on heart rate. Brain Stimul. 2018;11: 699–708. doi: 10.1016/j.brs.2018.04.004 29716843 PMC6536129

[18] Acharya UR, Joseph KP, Kannathal N, Lim CM, Suri JS. Heart rate variability: a review. Med Biol Eng Comput. 2006;44: 1031–1051. doi: 10.1007/s11517-006-0119-0 17111118

[19] Messina G, Vicidomini C, n. Viggiano A, Tafuri D, Cozza V, Cibelli G, et al. Enhanced parasympathetic activity of sportive women is paradoxically associated to enhanced resting energy expenditure. Auton Neurosci Basic Clin. 2012;169: 102–106. doi: 10.1016/j.autneu.2012.05.003 22682704

[20] Couck M De, Mravec B, Gidron Y. You may need the vagus nerve to understand pathophysiology and to treat diseases. Clin Sci. 2012;122: 323–328. doi: 10.1042/CS20110299 22150254

[21] Entschladen F, Drell TL, Lang K, Joseph J, Zaenker KS. Tumour-cell migration, invasion, and metastasis: navigation by neurotransmitters. Lancet Oncol. 2004;5: 254–258. doi: 10.1016/S1470-2045(04)01431-7 15050959

[22] Haensel A, Mills PJ, Nelesen RA, Ziegler MG, Dimsdale JE. The relationship between heart rate variability and inflammatory markers in cardiovascular diseases. Psychoneuroendocrinology. 2008;33: 1305–1312. doi: 10.1016/j.psyneuen.2008.08.007 18819754 PMC4266571

[23] Tsutsumi T, Ide T, Yamato M, Kudou W, Andou M, Hirooka Y, et al. Modulation of the myocardial redox state by vagal nerve stimulation after experimental myocardial infarction. Cardiovasc Res. 2008;77: 713–721. doi: 10.1093/cvr/cvm092 18065771

[24] Vlcek M, Radikova Z, Penesova A, Kvetnansky R, Imrich R. Heart rate variability and catecholamines during hypoglycemia and orthostasis. Auton Neurosci Basic Clin. 2008;143: 53–57. doi: 10.1016/j.autneu.2008.08.001 18793878

[25] Peuker ET, Filler TJ. The nerve supply of the human auricle. Clin Anat. 2002;15: 35–37. doi: 10.1002/ca.1089 11835542

[26] Widjaja D, Vandeput S, Taelman J, Braeken MA, Otte RA, den Bergh BR Van, et al. Accurate R peak detection and advanced preprocessing of normal ECG for heart rate variability analysis. 2010 Computing in Cardiology. 2010. pp. 533–536.

[27] Laborde S, Mosley E, Thayer JF. Heart Rate Variability and Cardiac Vagal Tone in Psychophysiological Research—Recommendations for Experiment Planning, Data Analysis, and Data Reporting. Front Psychol. 2017;8: 213. doi: 10.3389/fpsyg.2017.00213 28265249 PMC5316555

[28] Probe Q, Cardiovascular B. Power Spectrum Analysis of Heart Rate Fluctuation: A Quantitative Probe of Beat-to-Beat Cardiovascular Contr. 2014;213: 1–3. 10.1126/science.6166045 6166045

[29] Colzato LS, Ritter SM, Steenbergen L. Transcutaneous vagus nerve stimulation (tVNS) enhances divergent thinking. Neuropsychologia. 2018;111: 72–76. doi: 10.1016/j.neuropsychologia.2018.01.003 29326067

[30] Malik M, Bigger JT, Camm AJ, Kleiger RE, Malliani A, Moss AJ, et al. Heart rate variability. Standards of measurement, physiological interpretation, and clinical use. Eur Heart J. 1996;17: 354–381. 8737210

[31] Borges U, Laborde S, Raab M. Influence of transcutaneous vagus nerve stimulation on cardiac vagal activity: Not different from sham stimulation and no effect of stimulation intensity. PLoS One. 2019;14: 1–23. doi: 10.1371/journal.pone.0223848 31603939 PMC6788680

[32] Couck M De, Cserjesi R, Caers R, Zijlstra WP, Widjaja D, Wolf N, et al. Effects of short and prolonged transcutaneous vagus nerve stimulation on heart rate variability in healthy subjects. Auton Neurosci Basic Clin. 2017;203: 88–96. doi: 10.1016/j.autneu.2016.11.003 28017263

[33] Clancy JA, Mary DA, Witte KK, Greenwood JP, Deuchars SA, Deuchars J. Non-invasive Vagus Nerve Stimulation in Healthy Humans Reduces Sympathetic Nerve Activity. Brain Stimul. 2014;7: 871–877. doi: 10.1016/j.brs.2014.07.031 25164906

[34] Weise D, Adamidis M, Pizzolato F, Rumpf J-J, Fricke C, Classen J. Assessment of brainstem function with auricular branch of vagus nerve stimulation in Parkinson’s disease. PLoS One. 2015;10. doi: 10.1371/journal.pone.0120786 25849807 PMC4388709

[35] Yoshino K, Matsuoka K. Personal Adaptive Method to Assess Mental Tension during Daily Life Using Heart Rate Variability. Methods Inf Med. 2011;51: 39–44. doi: 10.3414/ME11-01-0027 22183777

[36] Koenig J, Jarczok MN, Ellis RJ, Hillecke TK, Thayer JF. Heart rate variability and experimentally induced pain in healthy adults: A systematic review. Eur J Pain (United Kingdom). 2014;18: 301–314. doi: 10.1002/j.1532-2149.2013.00379.x 23922336

[37] Aysin B, Aysin E. Effect of respiration in heart rate variability (HRV) analysis. Conference proceedings:. Annual International Conference of the IEEE Engineering in Medicine and Biology Society IEEE Engineering in Medicine and Biology Society Annual Conference. 2006. pp. 1776–1779. 10.1109/IEMBS.2006.260773 17946068

[38] Murray AR, Atkinson L, Mahadi MK, Deuchars SA, Deuchars J. The strange case of the ear and the heart: The auricular vagus nerve and its influence on cardiac control. Auton Neurosci Basic Clin. 2016;199: 48–53. doi: 10.1016/j.autneu.2016.06.004 27388046

[39] Haker E, Egekvist H, Bjerring P. Effect of sensory stimulation (acupuncture) on sympathetic and parasympathetic activities in healthy subjects. J Auton Nerv Syst. 2000;79: 52–59. doi: 10.1016/s0165-1838(99)00090-9 10683506

[40] Frangos E, Ellrich J, Komisaruk BR. Non-invasive access to the vagus nerve central projections via electrical stimulation of the external ear: FMRI evidence in humans. Brain Stimulation. Elsevier Ltd; 2015. doi: 10.1016/j.brs.2014.11.018 25573069 PMC4458242

[41] Wulsin LR, Horn PS, Perry JL, Massaro JM, D’Agostino RB. Autonomic imbalance as a predictor of metabolic risks, cardiovascular disease, diabetes, and mortality. J Clin Endocrinol Metab. 2015;100: 2443–2448. doi: 10.1210/jc.2015-1748 26047073

[42] Polak T, Markulin F, Ehlis AC, Langer JBM, Ringel TM, Fallgatter AJ. Far field potentials from brain stem after transcutaneous Vagus nerve stimulation: Optimization of stimulation and recording parameters. J Neural Transm. 2009;116: 1237–1242. doi: 10.1007/s00702-009-0282-1 19728032

[43] Joshi S, Li Y, Kalwani RM, Gold JI. Relationships between Pupil Diameter and Neuronal Activity in the Locus Coeruleus, Colliculi, and Cingulate Cortex. Neuron. 2016;89: 221–234. doi: 10.1016/j.neuron.2015.11.028 26711118 PMC4707070

[44] Ventura-Bort C, Wirkner J, Genheimer H, Wendt J, Hamm AO, Weymar M. Effects of Transcutaneous Vagus Nerve Stimulation (tVNS) on the P300 and Alpha-Amylase Level: A Pilot Study. Front Hum Neurosci. 2018;12: 202. doi: 10.3389/fnhum.2018.00202 29977196 PMC6021745

